# Reassessing Chronic Lyme Disease and Post-Treatment Lyme Disease Syndrome as Focal Infections

**DOI:** 10.7759/cureus.91246

**Published:** 2025-08-29

**Authors:** András Lakos, Gyöngyi Nagy, Zoltán Varga, Péter Rásonyi-Kovács, Sándor Hornok

**Affiliations:** 1 Outpatient Service, Centre for Tick-Borne Diseases, Budapest, HUN; 2 Laboratory/Outpatient Service, Centre for Tick-Borne Diseases, Budapest, HUN; 3 Dentistry, Navadent Dental Clinic, Budapest, HUN; 4 Otolaryngology, South-Pest Hospital Centre – National Institute for Infectology and Haematology, Budapest, HUN; 5 Department of Parasitology and Zoology, University of Veterinary Medicine Budapest, Budapest, HUN; 6 New Blood-Sucking Parasites and Vector-Borne Pathogens Research Group, Hungarian Research Network-University of Veterinary Medicine Budapest (HUN-REN-UVMB), Budapest, HUN

**Keywords:** chronic lyme disease, chronic parodontitis, chronic periodontitis, chronic tonsillitis, focal infection, lyme borreliosis, post treatment lyme disease syndrome (ptlds)

## Abstract

The complete clinical spectrum of Lyme borreliosis has been recognized for nearly 50 years, yet its diagnosis remains challenging due to the heterogeneity of symptoms. While many symptoms are likely nonspecific, a recurring cluster, including severe fatigue, brain fog, cognitive decline, memory impairment, joint and muscle pain, limb numbness, headaches, and low-grade fever, is often labeled in the scientific literature as post-treatment Lyme disease syndrome (PTLDS), and in the popular media as "chronic Lyme disease." Based on clinical experience and retrospective case analysis, this study hypothesizes that in many such cases, these persistent symptoms are not sequelae of Lyme borreliosis but manifestations of an undiagnosed focal infection, most commonly chronic tonsillitis or periodontal disease. The hypothesis is supported by the observation that the symptom profile of PTLDS is remarkably similar to that seen in focal infections, and by documented patient outcomes following treatment of these localized infections. This study compiles and analyzes clinical data to support the reinterpretation of PTLDS and “chronic Lyme disease” as misattributed focal infections in a subset of patients.

## Introduction

Lyme disease, also known in Europe as Lyme borreliosis, was first described as a complete clinical entity by Allen Steere and his colleagues in 1975, during their investigation of an arthritis outbreak in Connecticut [1]. In the first years following the discovery, publications began to emerge with titles and contents with the expression "the great imitator," previously used for syphilis [2]. The pathogen (*Borrelia burgdorferi*) does indeed resemble *Treponema pallidum*, as both belong to the order Spirochaetales. There are also similarities in the clinical presentation, as both diseases exhibit skin lesions, joint and neurological complications, acute and late-stage, chronic clinical forms. Since then, it has become widespread that any symptom or complaint for which we cannot find an explanation could be a consequence of Lyme disease, particularly believed to be part of its chronic manifestation.

In a publication by Lakos in 2017, discussing the distinction between true chronic Lyme disease and the so-called chronic Lyme disease, they could not find any evidence to support the presence of a chronic Borrelia infection [3]. This was followed by another pilot work by Lakos, suggesting that oral focal infection might be the underlying cause of “chronic Lyme disease” and/or post-treatment Lyme disease syndrome (PTLDS) [4]. The present study is a follow-up to these earlier works, aiming to demonstrate for the first time with 50 new cases that the clinical complaints and symptoms are essentially the same across the three conditions: “chronic Lyme disease”, PTLDS, and oral focal infection. The title of “chronic Lyme disease” is itself contradictory, as Lyme borreliosis can truly become a chronic illness. The most frequent symptom of Lyme disease, erythema migrans, can last for months. Other symptoms, such as Lyme arthritis, can last for years, and acrodermatitis chronica atrophicans for up to a decade, or even longer. However, "chronic Lyme disease" refers to a set of symptoms that have very little connection to Lyme borreliosis. The most comprehensive study to date [5], along with several others, found no evidence of Borrelia infection in these patients. Although many studies have attempted to formulate diagnostic criteria for "chronic Lyme disease", now often referred to as PTLDS, no consensus has been reached. Nevertheless, severe fatigue, brain fog, cognitive decline, memory impairment, joint and muscle pain, limb numbness, headaches, and low-grade fever are commonly mentioned in scientific publications [5]. These are mostly subjective complaints, typically without objective findings.

PTLDS develops in 10-20% of patients treated for Lyme disease, which corresponds to 100,000-200,000 new cases globally every year. Given the chronic nature of the syndrome, millions of people could be affected worldwide [6]. Despite the elusive nature of this symptom complex, we have noticed over 15 years of practice how homogeneous this group of patients is.

The objective of this study is to evaluate whether the symptom profiles of patients diagnosed with "chronic Lyme disease" or PTLDS are consistent with those observed in focal infections such as chronic tonsillitis or periodontal disease.

Focal infections, as defined in this study, are caused by partially closed abscesses that do not produce significant local symptoms but spontaneously drain, allowing chronic dissemination of inflammatory mediators or pathogens. These infections may develop in various organs, including the gallbladder, prostate, lungs, and ovaries, but are most commonly found in the oral cavity, particularly in the form of chronic tonsillitis or periodontal disease.

The hypothesis of this study is based on the clinical observation that the symptoms attributed to PTLDS closely mirror those commonly caused by such focal infections. Dental foci, including periapical abscesses, periodontal pockets, and chronic periodontitis, were frequently observed in patients referred to our center with suspected PTLDS.

This article was previously posted to the Preprint.org preprint server on June 27, 2025.

## Materials and methods

This study was conducted at the Center for Tick-Borne Diseases, Budapest, Hungary. Outpatients who presented to the Center for Tick-Borne Diseases between October 30, 2024, and May 12, 2025, diagnosed as suffering from "chronic Lyme disease" or PTLDS during the study period, and presented with (or reported) persistent symptoms commonly associated with these diagnoses (e.g., fatigue, brain fog, joint pain) for further diagnostic confirmation, were considered for the study.

All patients underwent clinical examination focused on identifying potential focal infections, including inspection of the oral cavity (tonsils, teeth, periodontal tissues). Patients in whom an obvious focal infection (e.g., chronic tonsillitis or periodontal disease) could not be identified were excluded from the study. A total of 50 patients were thus included in the study.

Patients completed a standardized questionnaire (see Appendix) indicating which symptoms they personally experienced. The questionnaire was compiled from complaints reported by our previous patients. No control group was used in this study. Sociodemographic characteristics, including sex and age, and routine laboratory test results (inflammatory markers: white blood cell count, CRP, blood sedimentation rate) were collected from the medical records.

Institutional criteria for chronic tonsillitis

Our institutional criteria for diagnosing chronic tonsillitis differ from those commonly found in the medical literature (Table 1).

**Table 1:** Our institutional criteria for diagnosing chronic tonsillitis

Criteria
Peaceful, delicate rose (not red) tonsils, and any of the following:
- Pus can be squeezed from the tonsils, or
- Pus can be seen coming from deep within; sometimes, small white pus clumps, or
- "Tonsil stones" the size of a pinhead or smaller, or even sizable, may be visible, or
- The tonsils are pitted, deeply fissured, or
- The tonsils are spectacularly asymmetrical, or
- Some parts or the entire tonsils are rounded, swollen, "inflated”

Clinical follow-up

**Figure 1:**
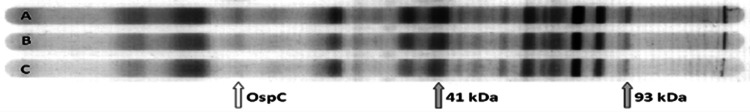
Serological regression Immunoblots (IgG) of a patient who suffered from acrodermatitis chronica atrophicans. Six years elapsed between the collection of the first (A) and the last (C) samples. It can be observed how intense the immune response develops in true chronic Lyme disease and how long it persists even after recovery. Although the decrease in antibody levels over time is minimal, it is nonetheless detectable — but only through direct comparison of archived and newly collected serum samples. This principle underlies the comparative immunoblot assay (COMPASS). Arrows indicate the antibody responses where the decrease is most impressive. Antibodies against the 93 kDa protein appear in infections lasting at least one year. OspC: surface protein C; 41 kDa: flagellar antigen Image Credit: Authors; this is an illustrative example from the authors' archives to demonstrate the capacity of the comparative immunoblot assay (COMPASS) to distinguish between past and active *Borrelia *infection. This is not part of the present study dataset, but is meant to support the rationale for considering "chronic Lyme disease"/post-treatment Lyme disease syndrome (PTLDS) patients as free of ongoing *Borrelia* infection.

**Figure 2:**
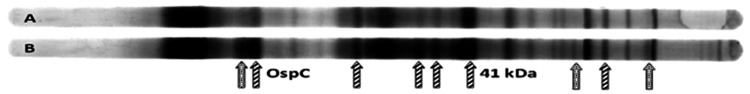
Serological progression Lyme arthritis lasting for a year: this always involves a robust IgG class immune response. Significant seroprogession is evident in the second sample (B), taken 48 days after the first sample (A) following unsuccessful antibiotic treatment (inflammation developed in the other knee joint after treatment). Arrows indicate the most significant changes. The patient recovered after receiving further antibiotic treatment. OspC: surface protein C; 41 kDa: flagellar antigen Image Credit: Authors; this is an illustrative example from the authors' archives to demonstrate the capacity of the comparative immunoblot assay (COMPASS) to distinguish between past and active *Borrelia* infection. This is not part of the present study dataset, but is meant to support the rationale for considering "chronic Lyme disease"/post-treatment Lyme disease syndrome (PTLDS) patients as free of ongoing *Borrelia* infection.

To determine if a patient has been cured of Lyme disease, a comparative immunoblot assay (COMPASS) is a sensitive procedure and effectively demonstrates microbiological recovery and, if applicable, the survival of Lyme bacteria after antibiotic treatment. This has been used for nearly 30 years. The essence of the test is to examine frozen stored older and newer serum samples side by side using the Western blot technique (Figures 1, 2) [7]. In this study, however, follow-up outcomes were not part of the analysis and were not systematically tracked.

Interestingly, even though we can detect pathogen survival through COMPASS, in most cases, this does not manifest clinically with symptoms or complaints. Based on this understanding, we developed a routine approach for these patients. If someone continues to experience the previously described symptoms after successful Lyme disease treatment, we look for signs of focal infection, starting with an examination of the mouth and throat.

Statistical analyses

Normal distribution of age-related data among patients was checked by the Kolmogorov-Smirnov test (https://www.socscistatistics.com/tests/kolmogorov/default.aspx). For comparison, data of 171 patients admitted to the Center of Tick-borne Diseases in Budapest and showing erythema migrans have been taken into account [8]. Student t-test was used to compare mean age, and Fisher's exact test (https://www.langsrud.com/fisher.htm) to compare the ratio of male and female patients according to symptom groups. Differences were regarded as significant if P < 0.05.

## Results

**Figure 3:**
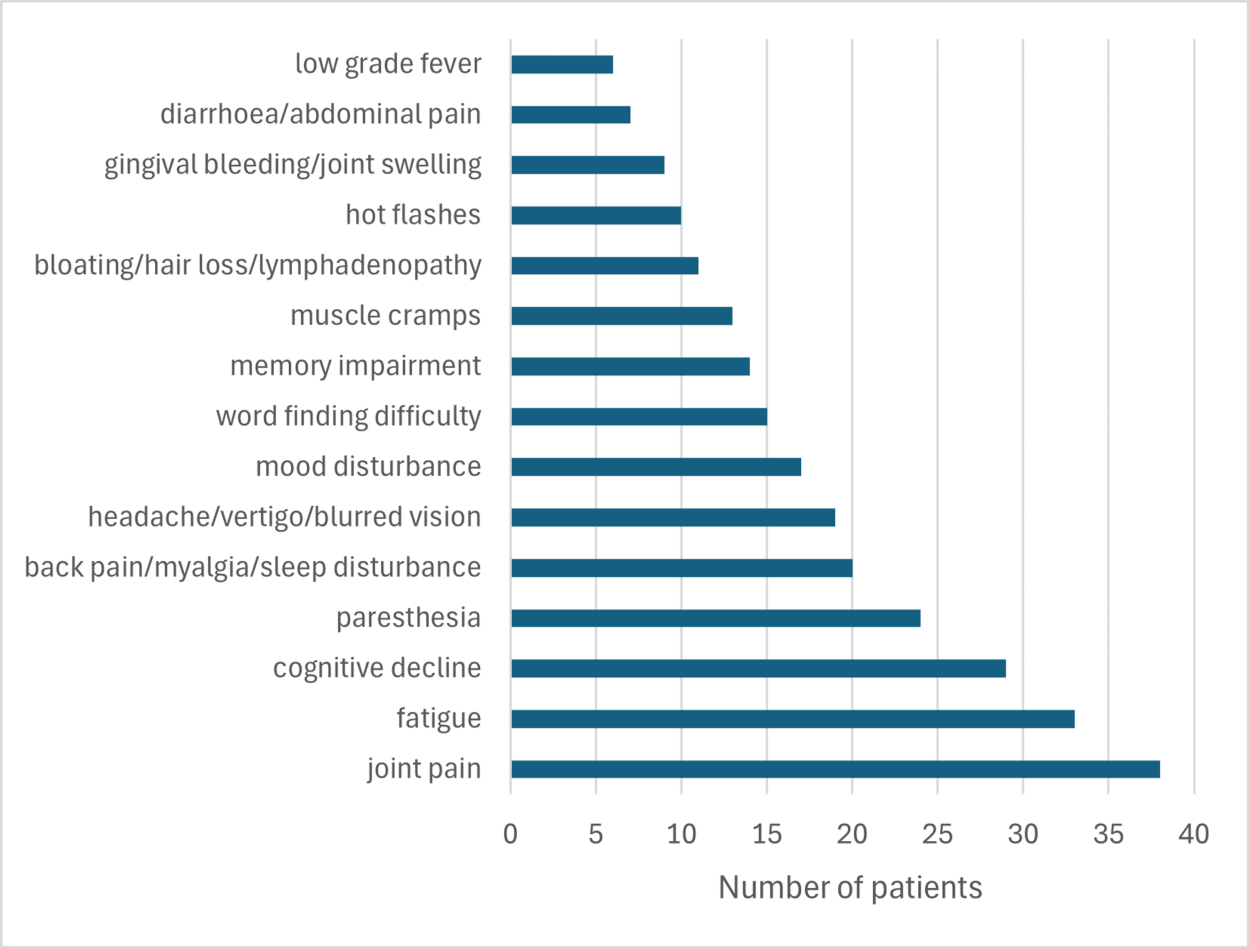
Summary of clinical symptoms and/or complaints of 50 patients included in the study

All the symptoms included in the questionnaire, self-identified by the 50 patients with a suspicion of "chronic Lyme borreliosis" or PTLDS, and recently having focal infection, are listed in Figure 3.

The patients referred to us commonly presented with similar symptoms, primarily fatigue, occasionally severe enough to cause inability to work, joint, bone, connective tissue, and muscle pain, general weakness, fatigue, sweating, non-rotating dizziness, "internal fever” (not indicated by a thermometer), uncertainty, low-grade fever, headaches, limb numbness or other sensory disturbances, muscle weakness, sleep disturbances, blurred vision. Objective symptoms were almost never present, and inflammatory markers in laboratory tests (white blood cell count, CRP, blood sedimentation rate) were also normal (data not shown).

The mean age of these 50 patients having focal infection was 43.14±12.33 years. This was significantly higher (P = 0.033) than the mean age of 171 patients diagnosed with erythema migrans in the comparative cohort from 1999-2021 (35.76±23.37 years) [8]. However, the ratio of males vs females did not differ significantly between these two groups; In the cohort from 1999-2021 showing erythema migrans, it was 76 men vs 95 women [8], whereas the patients with focal infection in the current study comprised 24 men vs 26 women (P = 0.75).

Patients having hair loss and diarrhea had the lowest mean age (34.8 and 38.4 years, respectively), and those with swollen joints had the highest (53 years). The mean age of patients having neurological symptoms was 42.03±12.17 years, higher but not significantly different from that in the group reported with gastrointestinal signs (36.72±15.68 years) (P = 0.063). However, the mean age of patients with gastrointestinal signs was significantly lower in comparison with the age of patients having musculoskeletal diseases, i.e., 44±12.36 years (P = 0.014). The mean age of patients with chronic tonsillitis (42.53±12.91 years) or dental foci (45.04±11.73 years) was similar (P = 0.46).

Considering the ratio of sexes among patients with focal infection, it was balanced in most symptom groups. However, hot flushes were more often characteristic of female (n=7) than of male patients (n=3), while this trend was the opposite in the case of sleeping disorder and muscle pain, in the case of which 1.5 times more men (n=12) developed this condition than women (n=8).

## Discussion

It is of utmost importance to have an explanation as to what might cause the symptoms of "chronic Lyme disease" to occur despite successful treatment of Lyme borreliosis. However, to the best of our knowledge, no one has yet reported deciphering what causes so many doctors to believe in the existence of "chronic Lyme disease." In our experience, as also shown here, almost every "chronic Lyme patient" has oral foci. Younger individuals tend to have chronic tonsillitis, while older individuals have dental foci, and some experience symptoms due to chronic sinusitis, although in the present study, this comparison did not yield a significant difference (probably owing to the limited number of cases). It is possible that many doctors do not address these issues because they do not thoroughly examine the oral cavity using appropriate tools.

**Figure 4:**
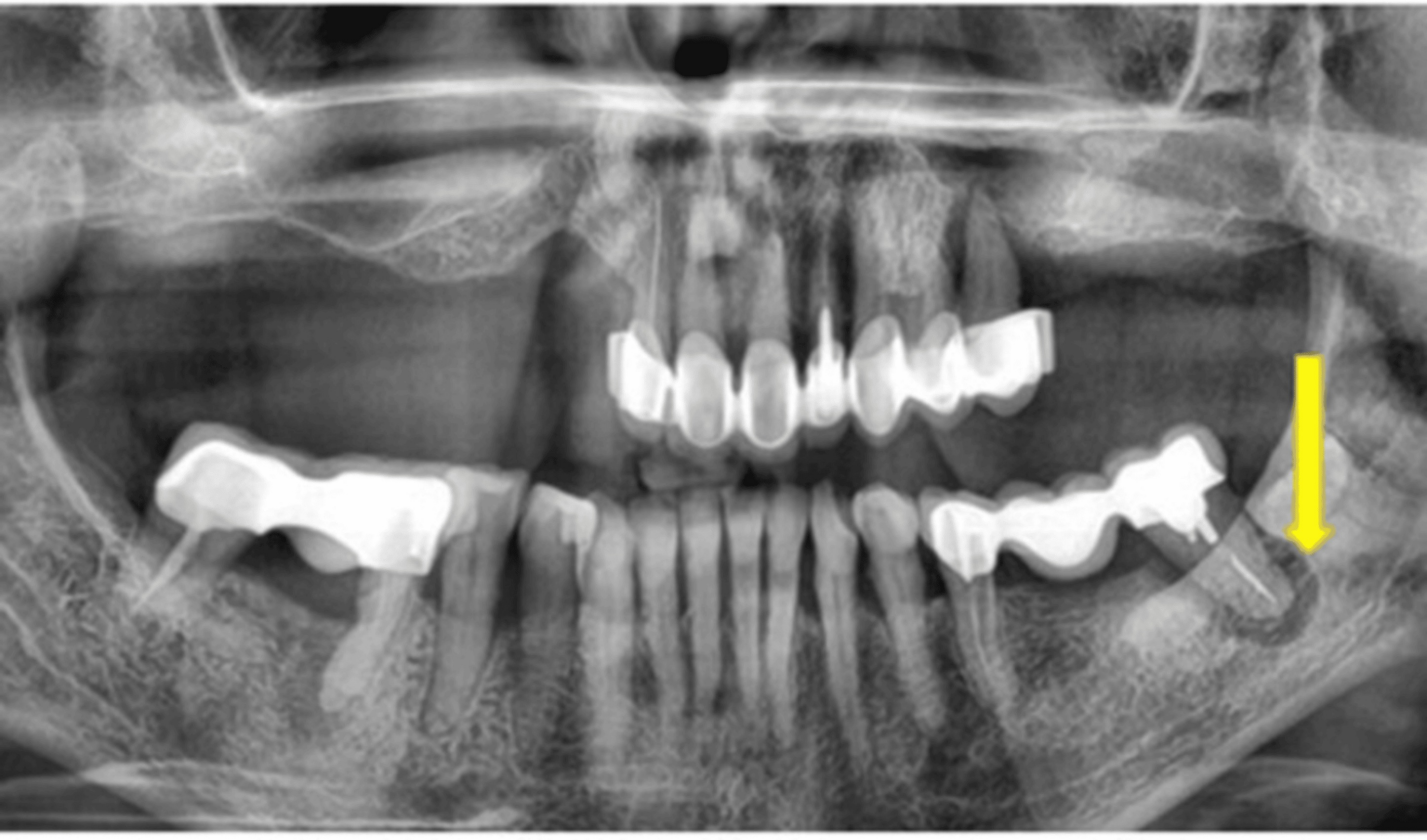
Giant periapical abscess (arrow); the tooth is only held by the bridge Image Credit: Dr. Zoltán Varga

**Figure 5:**
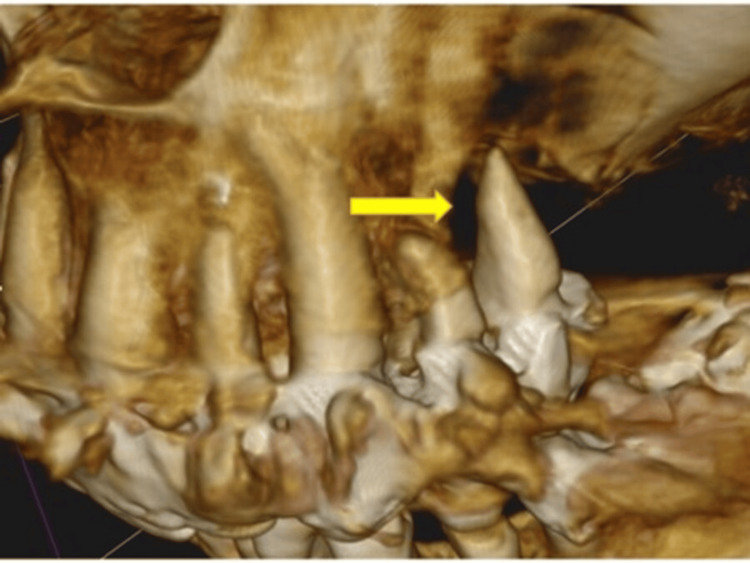
Giant periodontal pocket (arrow); the tooth is held by connected crowns Image Credit: Dr. Zoltán Varga

**Figure 6:**
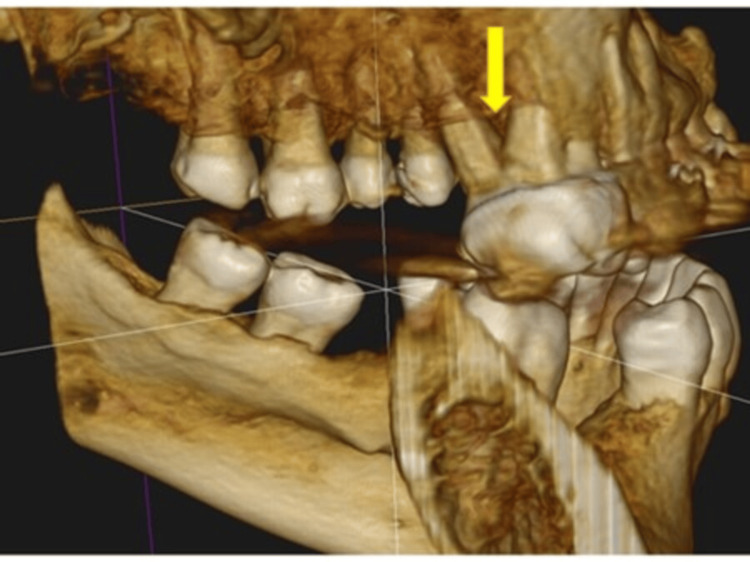
Periodontitis; Giant pocket (arrow) in a difficult-to-reach area between two tooth roots, not visible on the panoramic X-ray Image Credit: Dr. Zoltán Varga

**Figure 7:**
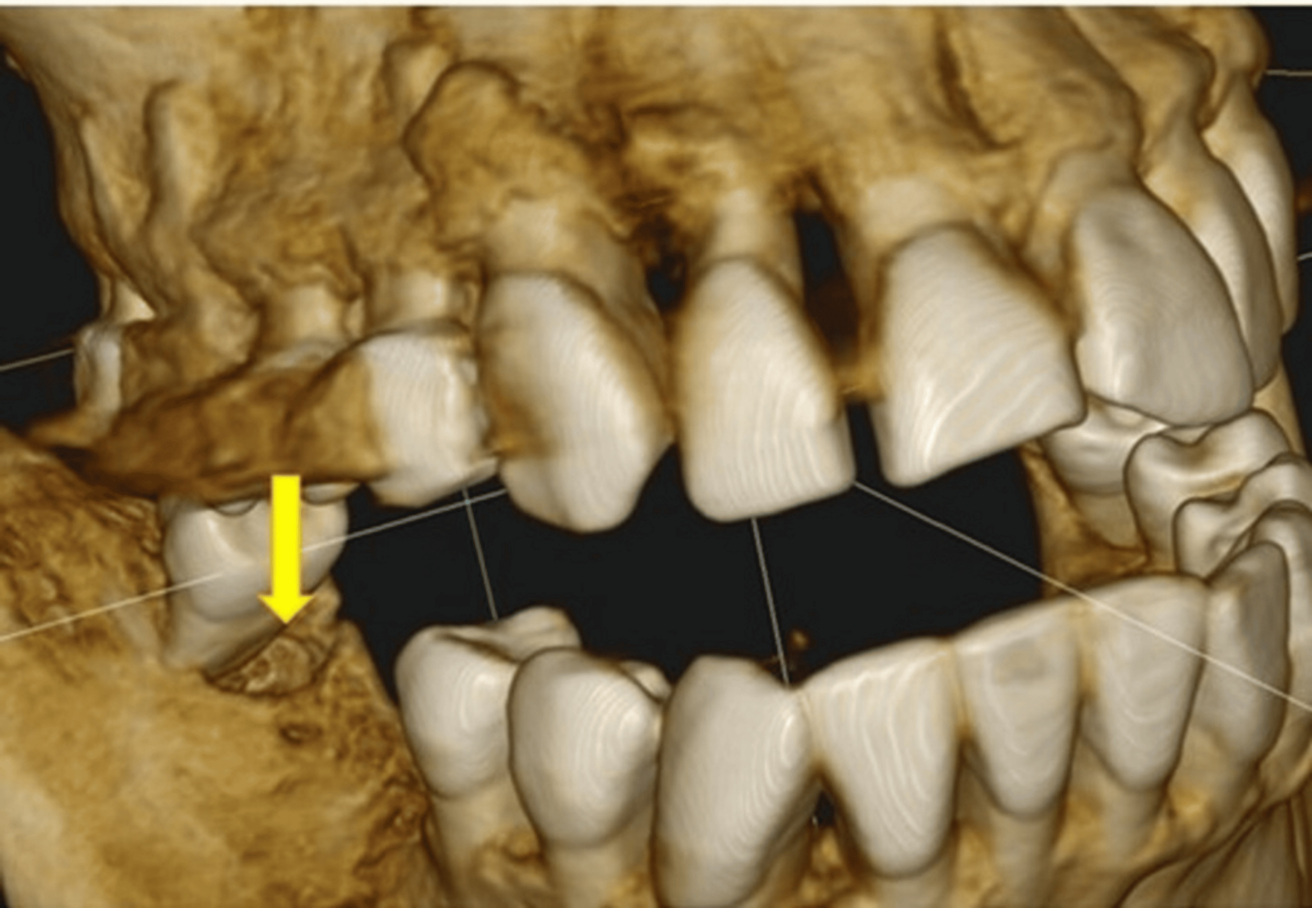
Enormous periodontal pocket (arrow) Image Credit: Dr. Zoltán Varga

**Figure 8:**
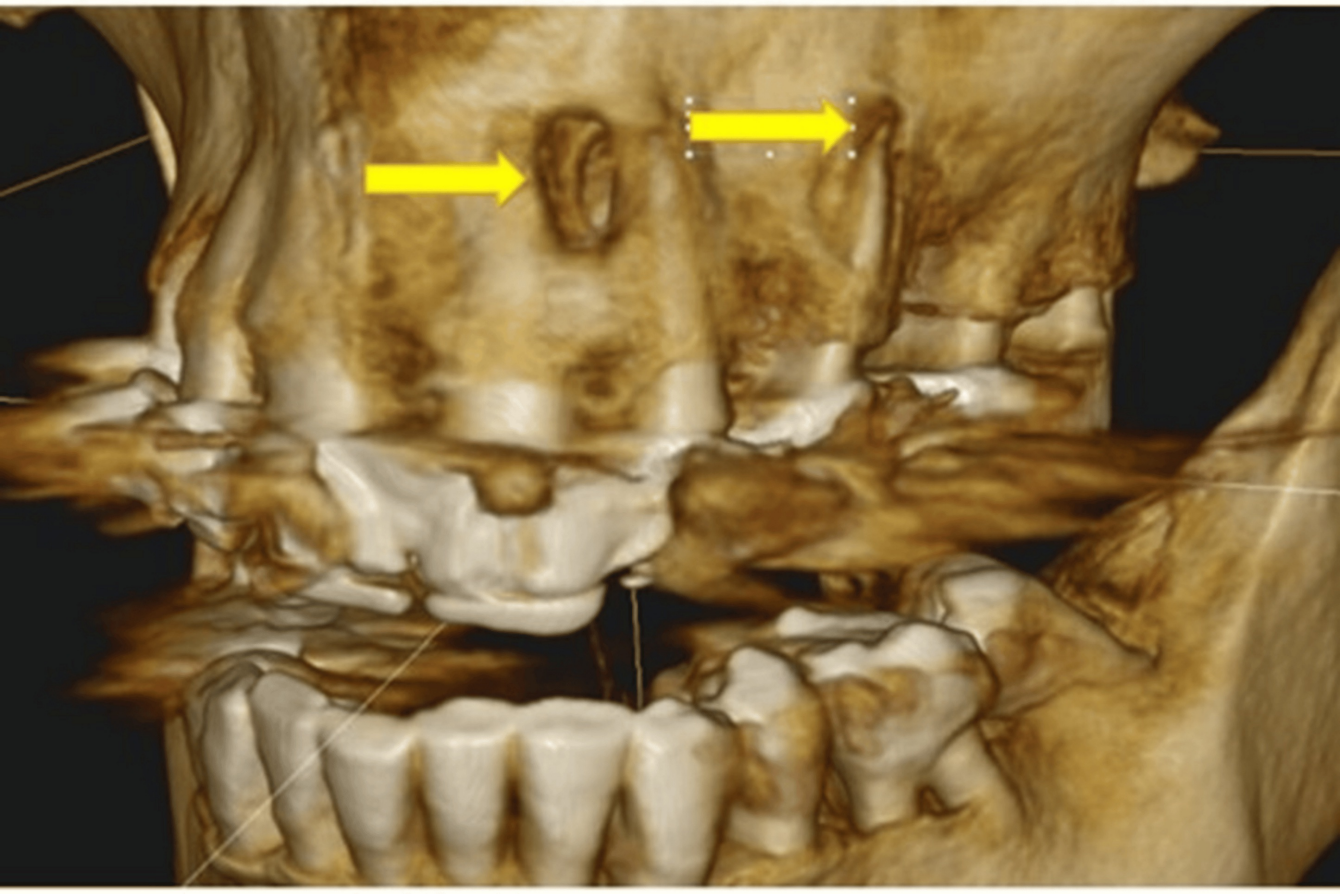
Periapical abscesses (arrows) NOTE: Pus in the tonsils could only be seen when gagging was induced with a spatula Image Credit: Dr. Zoltán Varga

**Figure 9:**
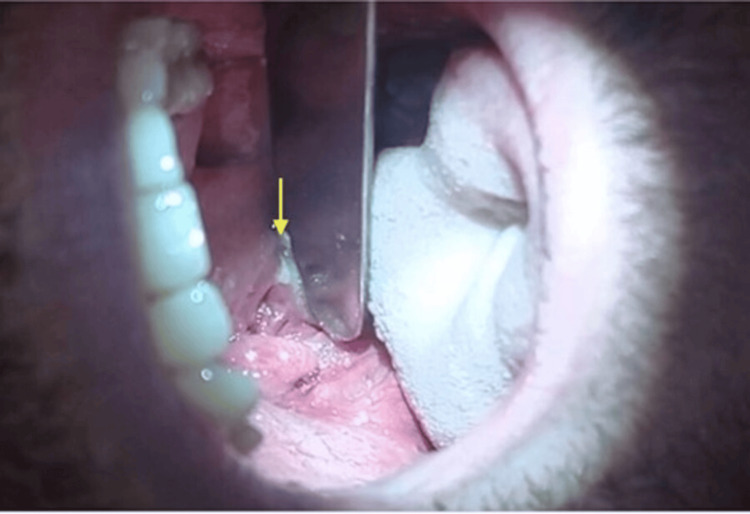
Chronic tonsilitis Pressing on the tonsil causes pus to spread onto the spatula (arrow). A tiny crater can be seen directly beneath the pus Image Credit: Dr. Péter Rásonyi-Kovács

**Figure 10:**
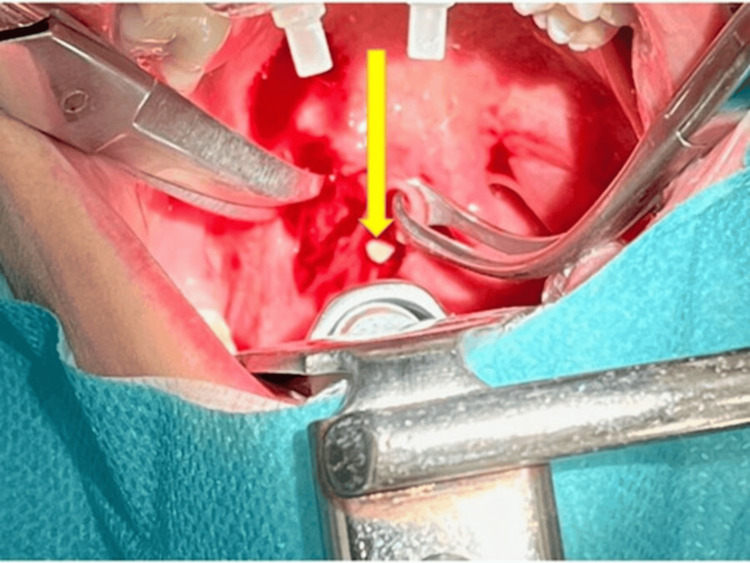
Surgical image Pus (arrow) only emerges when the surgeon applies pressure with the Kocher onto the tonsil Image Credit: Dr. Péter Rásonyi-Kovács

**Figure 11:**
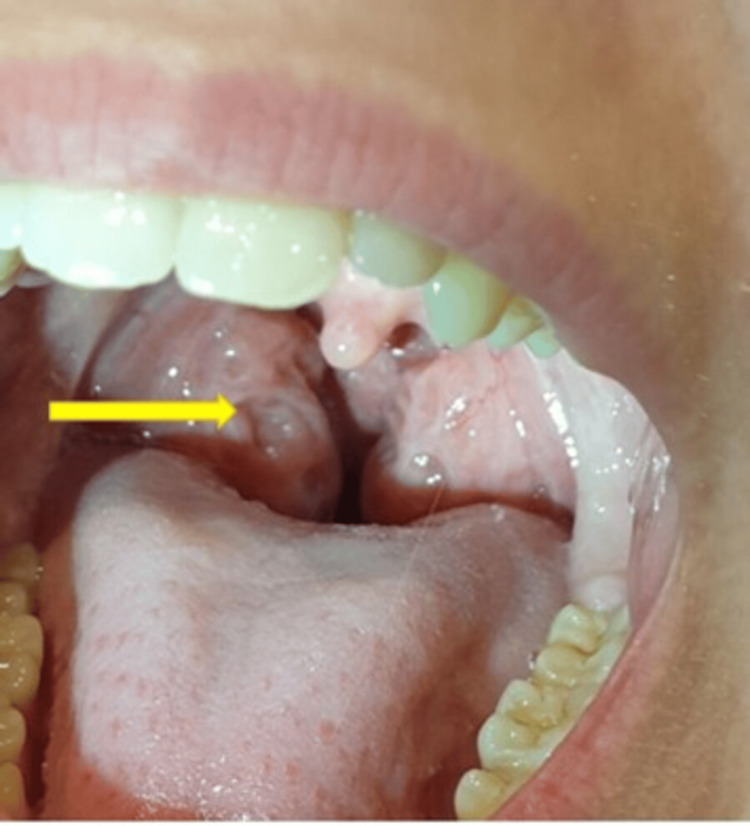
Spherical tonsils Nearly touching, scarred, cratered (arrow), almost spherical tonsils Image Credit: Gyöngyi Nagy

**Figure 12:**
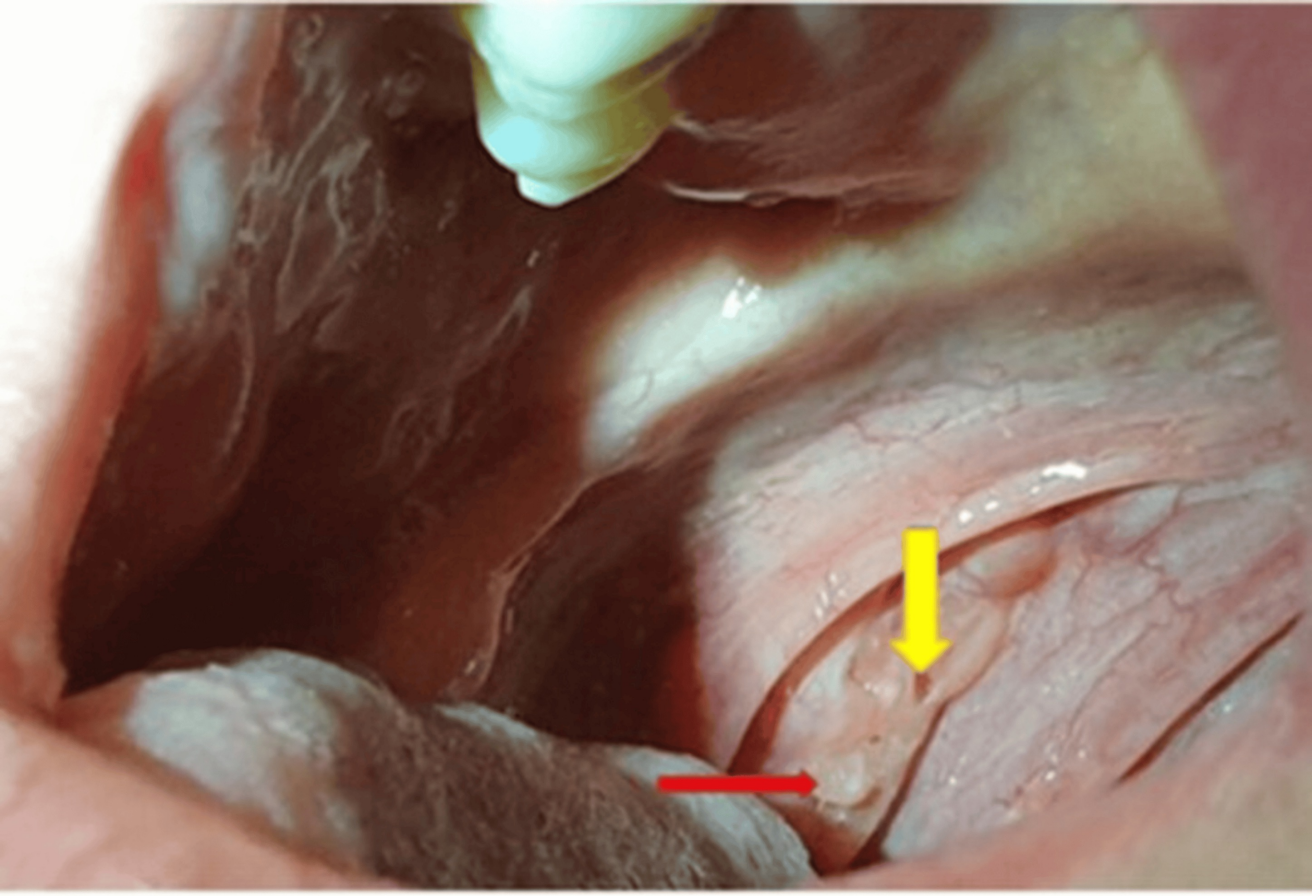
Chronic tonsilitis The almost completely atrophied tonsil barely emerges from the tonsillar fossae. The red arrow indicates a pus clump about to emerge, while the yellow arrow points to a crater showing recent pus discharge. Image Credit: Gyöngyi Nagy

It seems obvious that the foci originated much earlier than the Lyme borreliosis in each case. However, the foci are almost inactive, causing hardly any symptoms, although the presented images (Figures 4-12) show how serious the lesions can be. The role of *Borrelia* infection in activating the foci may be that the influx of numerous Lyme bacteria flooding the body at this time distracts the immune system, which previously controlled bacteria in the focus. In every article discussing "chronic Lyme disease" or PTLDS, severe fatigue is listed as a leading symptom [6]. Interestingly, this is hardly mentioned or not at all in articles discussing focal infections [9]. The second most common complaint among our patients was severe fatigue, often leading to disability. We believe the main cause of this is chronic inflammation, which occupies the immune system and consumes significant energy. Memory and sleep disturbances, brain fog, are common complaints. Since these patients feel very unwell, they visit doctors for years without receiving help, increasingly focusing on themselves, being sleep-deprived, and consequently experiencing memory impairment and many other subjective complaints. These gradually disappear after successful surgical intervention.

We see these same complaints in post-Lyme syndrome, which has been thoroughly discussed in the scientific medical literature. We consider it an important diagnostic sign because patients literally say the same thing: antibiotic treatment is temporarily effective, but after a few days, weeks, or months, the symptoms return. The success of antibiotic treatment confirms the misdiagnosis of Lyme disease, and so does the relapse, indicating how difficult it is to cure this disease. This prompts the treating physician to prescribe more and more antibiotics, increasingly longer, and more medications in parallel. The failure (and temporary success) of antibiotic treatment is an important diagnostic sign mentioned by many of our patients. The possible explanations are: (i) the metabolism of the focal bacteria is significantly reduced, and since antibiotics exert their effect by targeting metabolic pathways, their efficacy is limited under such conditions, (ii) since there is no effective antibiotic against every member of the extremely heterogeneous bacterial population, (iii) biofilm formation prevents the penetration of antibiotics [10], (iv) because there is no blood circulation in the wall of the abscess, there is nothing to deliver the active ingredients into the core of the focus [11]. These, particularly when disregarding professional recommendations, can have transient and moderate effects on the extraordinary and high-dose antibiotic combinations.

The International Lyme and Associated Diseases Society (ILADS) lists 91 symptoms that have been observed in "chronic Lyme disease" [12]. Among these, symptoms such as eyelid swelling, facial flushing, tinnitus, hearing loss, dizziness, torticollis, cogwheel rigidity (a hallmark of Parkinson’s disease), tremors, nocturia, Alzheimer’s disease, anetoderma, carpal tunnel syndrome, skin tumors, mitral insufficiency, mycosis fungoides, panuveitis, and ulcerative keratitis have never been observed by the authors in approximately 40,000 Lyme disease patients over the past four decades at the Center for Tick-borne Diseases in Budapest [unpublished data; authors' personal experience]. The problem with the plethora of listed symptoms is that Lyme disease is common enough, and other symptoms of different origins can easily occur alongside it as a confounding factor. Lyme disease foundations often consider so-called co-infections to be responsible for the development of "chronic Lyme disease" [12]. Anaplasma, babesia, and bartonella are the most commonly mentioned [13]. Based on improperly adjusted or incorrectly interpreted serological findings, a portion of patients is often treated for these pathogens [14].

In another questionnaire by a Lyme foundation in the United States, the symptoms of "chronic Lyme disease" are listed as follows: fatigue, headaches, joint and muscle pain, twitching, forgetfulness, cognitive impairment, sleep disturbances, heart problems, gastrointestinal issues, digestive disorders, neuropathies (numbness, tingling, altered sensation of hot or cold), depression, mood swings [15]. These symptoms are not truly specific and can sometimes occur in Lyme disease. However, for instance, headaches are almost never present in chronic forms, and gastrointestinal issues, while common, are not typically associated with Lyme disease. There are never any cardiac symptoms in chronic forms. Lyme carditis occurs with a prevalence of per thousand, involves atrioventricular block, always manifests in the first weeks of infection, and then resolves on its own, so there is no chronic variant. Lyme disease does not cause psychiatric disorders [16], although many publications claim the opposite [17]. Numerous reliable studies confirm that the aforementioned symptoms cannot be alleviated by any antibiotic treatment, no matter how prolonged or high the dose is [18-20].

"Chronic Lyme disease": focal infection with systemic consequences?

As shown in the current study, compared to the earlier group with erythema migrans [8], prolonged manifestation of focal infection is evident from the significantly more advanced age of patients in this study with focal infection (including both chronic tonsillitis and dental foci), as well as musculoskeletal diseases. According to Lohiya et al., periodontitis can be a source of various diseases in the cardiovascular, pulmonary, endocrine, musculoskeletal, central nervous, and reproductive systems [21]. Thus, dental focal infections may have serious systemic consequences. Periodontitis increases mortality and the risk of numerous systemic diseases such as diabetes mellitus, premature birth, and several cardiovascular diseases (atherosclerosis, heart attack, and stroke) [22-24]. According to a case-control, cross-sectional study, periodontitis doubles the risk of cardiovascular diseases and increases the risk of premature birth sevenfold [25]. It has been proven that there is a causal relationship between periodontitis and heart attack, adverse outcome of pregnancy, diabetes mellitus, and certain respiratory diseases [26]. Periodontal disease causes fibromyalgia [27]. A Hungarian study indicates how prevalent periodontal disease is: in Budapest and its surroundings, 16% of the population had completely healthy periodontium, while in rural areas, only 5-8% did [28]. Not only periapical abscesses count as focal infections, but also implants, gingivitis, and root-treated teeth. With genetic technology, numerous microbes can be isolated, half of which we cannot culture with currently available tools. Of course, not all detected bacteria have equal importance in maintaining the process, but we know very little about this.

Chronic tonsillitis as a focal infection

Despite the abundant literature on dental focal infections, we could not find any data in the available literature suggesting that others have noticed that chronic tonsillitis causes general symptoms. Yet, we observe the same symptoms behind chronic tonsillitis as those caused by periodontal diseases. However, there is some data supporting the connection between the two focal infections (chronic tonsillitis and dental infection). Genetic analysis of discharge from chronic tonsillitis isolated 42-110 species of bacteria, some of which were identical to those isolated from periodontitis [29]. Russian researchers also raised the connection between chronic tonsillitis and periodontitis [30].

**Figure 13:**
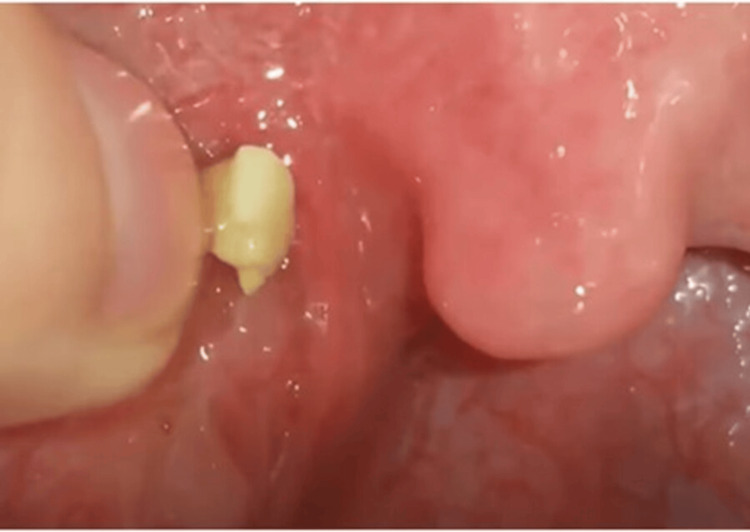
Tonsillolith being discarded Image Credit: Dr. James O'Donovan (https://www.youtube.com/shorts/xzaFGVMv-yE); used with permission

Tonsilolith is the extreme form of chronic tonsillitis. This is essentially calcified pus, which most otolaryngologists consider a harmless phenomenon. On the internet, one can find various tools with which patients can excavate these "stones" from their tonsils themselves (Figure 13). However, these stones are formed by a completely different mechanism than kidney or gallstones. In many cases, the body tries to isolate the inflammatory focus by depositing calcium, such as in tuberculosis or toxoplasmosis, and in certain parasitic infections (e.g., cysticercosis) as well. Tonsilolith consists of dead and living bacteria with minimal metabolism: fusobacteria, rods, and cocci are arranged in regular layers. Aerobic bacteria are found on the surface, creating the right environment for anaerobes located deeper [10]. Bacteria (and fungi) in dental foci are similarly structured, forming antibiotic-resistant biofilms [31]. These bacteria are practically uncultivable and resistant to antibiotics. It is a mixed flora, so it is another reason why it cannot be eradicated with a single or combined antibiotic, even with a prolonged course. Bacteria detected in throat swabs during culture are never the true pathogens but rather colonized microbes. The antibiotics prescribed against them are ineffective against the microbes residing in tonsil stones. Not only because antibiotics only affect actively metabolizing microbes, and not only because it's a mixed flora, but also because the bacterial colony forms a biofilm, preventing antibiotics from entering the abscess (because that's what a tonsil stone is: a calcified abscess). Tonsil stones are regularly expelled or located deep within the tonsils, so smaller ones can easily be overlooked. The surface of the tonsils is pale pink, with no signs of acute inflammation. Despite being a truly chronic inflammation, blood tests show no signs of inflammation; typically, all results are negative. All this misleads doctors and patients. Even though the diagnosis seems straightforward with tonsil compression, we see that otolaryngologists often interpret the picture differently and the course of action as well.

Limitations of the study

The main limitation of our study lies in the absence of any definitive clinical marker that would confirm whether the numerous subjective symptoms characteristic of "chronic Lyme disease" or PTLDS are always, or almost always, consequences of oral focal infections. We also lack data to support that oral foci consistently present with similar subjective complaints. The same applies to laboratory findings: despite the often severe or striking oral abscesses we encountered, inflammatory markers in blood tests were almost never elevated. In fact, significant inflammatory changes in laboratory parameters typically argue against the presence of a focal infection. Furthermore, this study did not include a control group, limiting the confidence in its findings.

Our reasoning would be much more convincing if we had data for all, or at least most, of our patients showing that they became symptom-free following the elimination of the oral focus. Unfortunately, recovery often takes several months, and it is likely that many of our patients do not follow our advice or do not receive care from competent specialists. As a result, we lack statistically analyzable data on recovery. Although we receive numerous grateful letters from patients, such testimonials do not allow us to assess the true rate of clinical recovery.

## Conclusions

The 50 cases and the reasoning presented in this study are meant as an initiative for larger-scale studies on this important topic. A multicentric study should be designed in which the diagnostic criteria for Lyme borreliosis, focal infections, and "chronic Lyme disease" or PTLDS are clearly defined. Doctors capable of accepting and adhering to these rules should be involved, together with experienced dentists and otolaryngologists. Patients participating in follow-up examinations are also needed, faithfully and credibly reporting the progression or regression of their subjective complaints, as there is no way to detect objective parameters due to the nature of the issue.

## Appendices

Appendix A: symptom questionnaire

Please circle the symptoms or complaints that applied to you at presentation:

Sweating, hot flashes, sleep disturbances, bleeding gums, fatigue, decreased performance, low-grade fever, headache, dizziness, numbness, memory problems, visual disturbances, floaters in the eyes, word-finding difficulties, diarrhoea, bloating, abdominal pain, mood swings, lower back pain, joint pain, joint swelling, muscle pain, muscle cramps, nerve pain, hair loss, swollen lymph nodes, antibiotic treatment provides temporary relief.
